# Common Echocardiographic Diagnoses in Patients at Tertiary Care Center: An Observational Study

**DOI:** 10.31729/jnma.8794

**Published:** 2024-11-30

**Authors:** Prabha Chapagain Koirala, Richa Koirala, Lochan Karki, Milan Khadka, Prasanna Bahadur Amatya, Binayak Gautam, Samir Poudel, Kunjang Sherpa, Saroj Ghimire, Rajendra Bhandari, Pragya Koirala

**Affiliations:** 1 Department of Cardiology, National Academy of Medical Sciences, Bir Hospital, Mahaboudha, Kathmandu, Nepal; 2 B.P Koirala Institute of Health Sciences, Buddha Road, Dharan, Nepal; 3 Department of Internal Medicine, National Academy of Medical Sciences, Bir Hospital, Mahaboudha, Kathmandu, Nepal; 4 Kanti Children's Hospital, Maharajgunj, Kathmandu, Nepal

**Keywords:** *diastolic dysfunction*, *doppler*, *left ventricular hypertrophy*, *systolic dysfunction*, *transthoracic echocardiography*

## Abstract

**Introduction:**

Echocardiography is an indispensable tool for the diagnosis and management of cardiac patients. It is important to know the spectrum of cardiac abnormalities detected by echocardiography for the proper management of patients. The aim of this study was to evaluate the echocardiographic diagnosis of patients at a multispecialty tertiary care hospital and find out the prevalence of various cardiac diseases diagnosed via echocardiography.

**Methods:**

This was a retrospective cross sectional study carried out at the Echocardiography lab of National Academy of Health Sciences. Records of all patients who had done Echocardiography from February 2024 to April 2024 were analyzed. Ethical approval was obtained from Institutional Review Committee (Reference number:1231/2080/81).

**Results:**

Out of 3593 cases, there were 1750 (48.71%) female and 1843 (51.29%) male. Normal Echocardiographic study was seen in 1184 (32.95%). Mean age was 56.13±17.38 years. Left ventricle diastolic dysfunction was seen among 1428 (39.74%), followed by Tricuspid Regurgitation 1026 (28.55%) and, Pulmonary Hypertension 497 (13.83%). Other findings were, left ventricular hypertrophy 204 (5.67%), Left ventricular systolic dysfunction 165 (4.59%), Rheumatic heart Disease 102 (2.83%) and, congenital heart disease was the lowest documented finding, seen in 30 (0.83%).

**Conclusions:**

Most common diseases diagnosed by echocardiography in our study were, diastolic dysfunction, tricuspid regurgitation and pulmonary hypertension.

## INTRODUCTION

Transthoracic echocardiography (TTE) is one of the most commonly used noninvasive initial imaging modalities in assessing acute and chronic cardiac conditions. Echocardiography (ECHO) is used to study the anatomy of the heart, including valves, chamber size, wall motion, masses, pericardial fluid. Doppler ECHO can assess the severity of valvular regurgitation, gradients across valves or between cardiac chambers and detect intra-cardiac shunts.^1^ Assessing left ventricular (LV) function is one of the common reason for requesting ECHO.^2^ Echocardiography represents the gold standard for assessment of LV systolic dysfunction.^3^ Doppler combined with two dimensional echocardiography is the best noninvasive method to evaluate diastolic dysfunction and its severity.^4^ Echocardiography is an important tool in recognizing acute rheumatic fever, Rheumatic Heart Disease (RHD), subclinical RHD, and its subsequent complication.^5^

While some studies regardiong ECHO findings have been conducted in Nepal previously, the decade-long gap highlights the need for updated information on the current patterns of cardiac disease prevalence.^6,7^ Therefore, this study aims to find the proportion of various echocardiographic diagnoses. The data obtained from this study will provide a baseline for conducting other research in cardiac diseases.

## METHODS

This was a hospital-based retrospective observational study. This study was done at National Academy of Health Sciences (NAMS) which is a tertiary care center at Kathmandu, Nepal. This being a multispecialty government hospital, it serves as a referral center for patients all around Nepal. Records of all patients who underwent echocardiography during the three months from February to April 2024 , were analyzed. The record of single patient could have more than one echocardiographic finding. Ethical approval was taken from Institutional Review Committee of NAMS (Reference number: 1231/2080/81).

Dilated cardiomyopathy was diagnosed if there was left ventricular or biventricular dilatation and, impaired cardiac contraction with decreased systolic function, as measured by left ventricular ejection fraction (LVEF) excluding, ischemic, valvular, hypertensive or congenital causes.^8^ Ischemic cardiomyopathy was diagnosed by assessing the regional wall motion abnormality on the basis of a documented history of myocardial infarction or acute coronary syndrome.^9^

For the estimation of pulmonary arterial hypertension (PAH), pulmonary artery systolic pressure (PASP) was calculated based on a simplified Bernoulli equation applied to peak tricuspid regurgitation velocity (TRV), PASP = 4 (TRV)^2^ + Right Arterial Pressure (RAP).^10^ Empirically 5 mm Hg was added when Tricuspid Regurgitation Peak Gradient (TRPG) was 30-39 mm Hg and, 10 mm Hg was added when TRPG was 40 mm Hg or more. The PAH was classified as follows: PASP 3549 mmHg as mild PAH, 50-70 mmHg as moderate PAH and, 70 mm Hg or more as severe PAH.^11^

Pericardial effusion was classified as: trivial as seen only in systole, mild <10 mm, moderate 10-20 mm and severe <20 mm.^12^ Left Ventricular (LV) systolic function was diagnosed as follows, normal: Left Ventricular Ejection Fraction (LVEF) 50-70 %, mild dysfunction: LVEF 40-49 %, moderate dysfunction: LVEF 30-39% and severe dysfunction: LVEF less than 30%.^13^ Rheumatic Heart Disease (RHD) was diagnosed by applying the 2012 World Heart Federation Echocardiographic criteria. ^14^

For the determination of LV diastolic dysfunction, the transmittal early diastolic rapid filling (E-wave) and atrial contraction late filling (A-wave) velocities, E/A ratio was calculated. For tissue Doppler imaging, the mitral annulus velocity was obtained with a 2 mm sample volume placed at the septal side and lateral side of the mitral annulus. Left ventricular diastolic dysfunction was considered to be present if any of the following findings were seen: E/A ratio < 1 or > 2, along with tissue Doppler septal e velocity <8 mm or lateral e velocity <10 mm or septal E/e ratio > 15. ^15^ Left Ventricular hypertrophy was diagnosed when there was posterior wall thickness of 1.0 cm.^8^

Color Doppler imaging was used for the detection and quantitation of mitral, tricuspid and aortic regurgitation. Both apical four chamber view and PLAX view was used.

The Statistical Analysis was done using the IBM SPSS Statistics for Windows, version 23 (IBM Corp., Armonk, N.Y., USA). Descriptive statistics were used to describe and summarize the data. Categorical variables were analyzed as percentage, and continuous variable with normal distribution were presented as mean±SD.

## RESULTS

Out of 3593 cases, there were 1750 (48.71%) female and 1843 (51.29%) male. The mean age of the study population was 56.13±17.38 years. Out of the total population, 84 (2.34%) belonged to the age group 0-20 years, 550 (15.31%) to 20-40 years, 1272 (35.40%) to 40-60 years and 1687 (46.95%) to the age group of 60 years and greater.

Normal ECHO study was seen in 1184 (32.96%) of cases. Left ventricular diastolic dysfunction was observed in 1428 (39.74%), Tricuspid Regurgitation (Non-RHD) in 1026 (28.55%) and, Pulmonary Hypertension (including RHD patients) in 497 (13.83%), (Table 1).

Mean pulmonary artery pressure recorded was 49.42 ± 14.46 mmHg. Severe LV systolic dysfunction was seen in 35 (21.21%), severe Tricuspid Regurgitation (TR) in 124 (12.09%), severe PAH in 55 (11.07%) patients. Among RHD patients, Mitral Regurgitation (MR) was 85 (83.33%), Tricuspid Regurgitation (TR) 59 (57.84%), Aortic Stenosis (AS) 33 (32.35%) and Mitral Stenosis (MS) 25 (24.50%). Among Congenital Heart Disease (CHD), Atrial Septal Defect (ASD) was 19 (63.33%), bicuspid aortic valve was 6 (20.00%) and Ventricular Septal Defect was 4 (13.33%), (Table 2).

Out of RHD lesion Mitral Regurgitation (MR) was 85 (83.33%) and Tricuspid Regurgitation was 59 (57.84%), Table 3.

**Table 1:** Common ECHO diagnosis in male and female (n=3593).

ECHO Diagnosis	Number of male patients n (% within male)	Number of female patients n (% within male)	Total number of patients
Normal ECHO	614 (33.31)	570 (32.57)	1184 (32.96)
ECHO with Findings	1229 (66.69)	1180 (67.43)	2409 (67.04)
Ischemic Cardiomyopathy	33 (1.78)	22 (1.25)	55 (1.53)
LV Diastolic Dysfunction	760 (41.23)	668 (38.17)	1428 (39.74)
LV Systolic Dysfunction	96 (5.21)	69 (3.94)	165 (4.59)
Dilated Cardiomyopathy	51 (2.76)	42 (2.0)	93 (2.58)
Pericardial Effusion	36 (1.95)	32 (1.82)	68 (1.89)
Pulmonary Hypertension	229 (12.42)	268 (15.31)	497 (13.83)
Rheumatic Heart Disease	40 (2.17)	62 (3.54)	102 (2.83)
Congenital Heart Disease	17 (0.92)	13 (0.74)	30 (0.83)
Left Ventricle Hypertrophy	134 (7.27)	70 (4.00)	204 (5.67)
Tricuspid Regurgitation not including RHD	502 (27.23)	524 (29.94)	1026 (28.55)

Echo= Echocardiogram, LV = Left ventricle

**Table 2:** Severity pattern of various ECHO diagnosis (n=3593)

Echocardiographic Diagnosis	Category	Number of Male Patients n (% within Male)	Number of Female Patients n (Percentage % within Female)	Total number of patients
LV Systolic Dysfunction, N=165	Mild LVEF (40-49%)	45 (46.88)	34 (49.28)	79 (47.89)
	Moderate LVEF (39-30%)	28 (29.16)	23 (33.33)	51 (30.90)
	Severe LVEF <30%	23 (23.96)	12 (17.39)	35 (21.21)
	Total patients	96 (100)	69 (100)	165(100)
Tricuspid Regurgitation not including RHD, N=1026	Mild	385 (76.70)	379 (72.33)	764 (74.46)
	Moderate	64 (12.75)	74 (14.12)	138 (13.45)
	Severe	53 (10.55)	71 (13.55)	124 (12.09)
	Total patients	502 (100)	524 (100)	1026 (100)
Pulmonary Hypertension including RHD, N= 497	Mild (35-49 mm Hg)	150 (65.50)	156 (58.21)	306(61.57)
	Moderate (50-69 mm Hg)	52 (22.70)	84 (31.34)	136(27.36)
	Severe (70 mm Hg and more)	27 (11.80)	28 (10.45)	55(11.07)
	Total patients	229(100)	268(100)	497(100)
Pericardial effusion, N=68	Mild	24 (66.67)	21 (65.62)	45(66.18)
	Moderate	10 (27.78)	3 (9.38)	13(19.12)
	Large	2 (5.55)	6 (18.75)	8(11.76)
	Impending Tamponade	0 (0)	2 (6.25)	2(2.94)
	Total	36(100)	32(100)	68(100)
Congenital Heart Disease, N=30	ASD	11 (64.71)	8 (61.54)	19(63.33)
	VSD	2 (11.76)	2 (15.39)	4(13.33)
	Ebstein Anomaly	1 (5.88)	-	1(3.34)
	Bicuspid aortic valve	3 (17.65)	3 (23.07)	6(20.0)
	Total patients	17 (100)	13 (100)	30(100)

ECHO= Echocardiogram, LV= Left ventricle, RHD = Rheumatic heart disease, LVEF = Left ventricular ejection fraction, ASD= Atrial septal defect, VSD= Ventricular septal defect.

**Table 3:** Pattern of different RHD lesion among patients, n=102 (female=62, male=40).

RHD lesion	Number of male patients (%within male)	Number of female patients (% within female)	Total patients
MS	11 (27.50)	14 (22.58)	25 (24.50)
MR	33 (82.50)	52 (83.87)	85 (83.33)
AR	16 (40)	17 (27.41)	33 (32.35)
AS	4 (10)	1 (1.61)	5 (4.90)
TR	22 (55)	37 (59.67)	59 (57.84)

RHD = Rheumatic Heart Disease, MS = Mitral Stenosis, MR = Mitral Regurgitation, AR = Aortic Regurgitation, AS = Aortic Stenosis, TR = Tricuspid Regurgitation

## DISCUSSION

This study consisted of large sample size (3593) with almost equal gender distribution (51.29 % male vs 48.71 % female). The objective of this study was to find the common conditions diagnosed by using transthoracic echocardiography in a multispecialty hospital. Diastolic dysfunction was the highest documented diagnosis in our study (39.74%) which is consistent with various other studies done in Nepal that have reported high prevalence of diastolic dysfunction.^6,7^ In the study done in Dhulikhel Hospital, LVDD was seen in the range of 20.6% to 57.14% across patients of different age groups. ^6^ Another study done in Nepal Medical College, reported LVDD in 36.1% of cases.^7^ Our study population had a large percentage of elderly patients, with 47% of them belonging to age group 60 years and greater which might account for the higher prevalence of diastolic dysfunction in our study.^16,17^

Being a multispecialty center, COPD with cor-pulmonale patients are frequently referred for ECHO screening. Owing to this, TR and Pulmonary Hypertension were the second and third most commonly documented findings. Tricuspid regurgitation (excluding RHD patients) was identified in 28.55 % of cases, which is relatively low compared to another study, that reported, tricuspid regurgitation (TR) in 68% of the population.^18^ Yet another study done in Nepal reported mild TR in 53% of patients.^7^

Pulmonary hypertension (PAH) was seen in 13.83 % of patients which is higher than that of a study carried out in Italy that reported 4.3% PAH in an unselected population.^19^ This can be attributed to our hospital setting that included both, outpatients and indoor patients

The LV systolic dysfunction was seen in 165 (4.59 %) of cases, low as compared to other studies that have documented LV systolic dysfunction in 10.56% and 13.9% patients respectively.^6,7^ In our study, the most common cause was dilated cardiomyopathy 60%, followed by ischemic heart disease 30% and, 10% accounted for other causes. This result is similar to that of another study which also reported dilated cardiomyopathy 47.7% followed by ischemic heart disease 13.6%, as the significant cause of heart failure.^20, 21^ Similarly, another study done in Nepal reported ischemic heart disease as the cause of LV systolic dysfunction in 12.80% to 25.87% population across different age groups.^6^

Left ventricular hypertrophy was seen in 5.67 % of patients. In a developing country like ours, there is a huge burden of hypertension, diabetes mellitus.^22^ Our study population consisted of patients referred from other departments, with comorbidities like chronic kidney disease, stroke etc. These factors might explain the prevalence of LV hypertrophy seen in our population. It also indicates that blood pressure is not properly controlled in our patients. According to the Step Survey Nepal 2019, the prevalence of hypertension was 24.5%. Among them, 78.8% adults were unaware about their raised BP, 11.7 % were not taking any treatment despite being aware of their raised BP, 5.4% were on treatment, but their BP was not controlled and, only 4.1% were on treatment and also had their BP controlled.^22^

In our study, RHD was present in 2.83% of cases, this might not reflect the actual picture as most of the RHD patients visit cardiac centers, which is another hospital specialized for cardiac care only.

Congenital heart disease was found very low in our study, seen in 0.83% patients, as our hospital lacks pediatric department and most of the children receive treatment in pediatric hospitals and with pediatric cardiologists. Despite this, most common lesion documented was ASD because clinical manifestations of ASD begin in adulthood.^23^ Similar to this, the study done in Dhulikhel Hospital also reported ASD as the most common congenital heart disease. ^6^

Since ECHO records were analyzed to generate the data, we do not have information regarding the indications for performing ECHO in the patients, which could have made the results more comprehensive.

## CONCLUSIONS

Most common diseases diagnosed by echocardiography in our study were, diastolic dysfunction, tricuspid regurgitation and pulmonary hypertension. Dilated cardiomyopathy was the common cause of heart failure in our setting.
